# Intersectoral communication amongst healthcare providers regarding care plans: a scoping review

**DOI:** 10.1177/26323524221092457

**Published:** 2022-04-21

**Authors:** Jodi Langley, Nikolas Jelicic, Taylor G. Hill, Emily Kervin, Barbara Pesut, Wendy Duggleby, Grace Warner

**Affiliations:** Faculty of Health, Dalhousie University, 5968 College Street, Halifax, NS B3H 4R2, Canada; School of Health and Human Performance, Dalhousie University, Halifax, NS, Canada; Department of Psychology and Neuroscience, Dalhousie University, Halifax, NS, Canada; Healthy Populations Institute, Dalhousie University, Halifax, NS, Canada; Faculty of Medicine, Dalhousie University, Halifax, NS, Canada; Faculty of Nursing, The University of British Columbia, Kelowna, BC, Canada; School of Nursing, University of Alberta, Edmonton, AB, Canada; School of Occupational Therapy, Dalhousie University, Halifax, NS, Canada

**Keywords:** advanced care plan, healthcare communication, intersectoral communication, palliative care, primary care

## Abstract

Palliative care has become an increasingly important public health issue due to the rising acceptance of implementing a health promoting palliative care approach. To explore communication pathways that would facilitate implementation of this approach, we conducted a scoping review examining communication and enactment of care plans for older adults with life-limiting illnesses across health, social and community sectors. We used a scoping review methodology to map the current literature on communication plans between primary care and other sectors (community, health, and social). Five databases were searched MEDLINE (ovid), CINAHL (EBSCO), EMBASE (Elsevier), PsychInfo (EBSCO), and Scopus. The database search identified 5,289 records, after screening and hand-searching a total of 28 articles were extracted. Three major themes were determined through the records: (1) the importance of professional relationships across sectors, (2) the importance of community navigators in sharing the care plan, and (3) and creating comprehensive and multidisciplinary care plans. Findings suggested that enacting quality care plans is important to healthcare providers; the use of an electronic health records system can be useful in ensuring that all healthcare and community systems are in place to aid patients for better community-based care. Community navigators were also key to ensure that plans are communicated properly and efficiently. Further research is needed to determine how having a clear and properly implemented communication system for a healthcare system could facilitate community sector involvement in implementing care plans.

## Introduction

The Canadian population is aging. In 2014, 15.6% of Canada’s population was aged 65 or older.^1^ By 2030, there will be 9.5 million seniors living in Canada, comprising 23% of its population.^1^ In addition to age, chronic disease rates in Canada are also increasing. It is estimated that one in five Canadians over the age of 20 have a chronic disease, while four out of five are at risk of diagnosis of a chronic disease.^2^ Every year, over 150,000 Canadians die from such diseases, and, unfortunately, this fraction of Canada’s population is only expected to grow larger as it ages.^2,3^ These trends, in both aging and chronic disease rates, have been paralleled across the globe, presenting healthcare systems with new challenges in providing impactful patient care.^4^ In addressing these challenges, interdisciplinary medical teams worldwide are initiating, exploring, and providing palliative approaches to care.^5–7^

The palliative approach to care aims to improve quality of life and reduce suffering for people living with life-limiting conditions.^8^ Typical provisions of the palliative approach include pain and symptom management, psychological, social, emotional, spiritual, and practical support, as well as added support for caregivers during the illness and after the death of the person in their care.^8^ A critical aspect of the palliative approach is its emphasis on patient- and family-centeredness, necessitating respect for and solicitation of the voices, opinions, and feelings of those with life-limiting illness and their families.^8^

It is important to note that a palliative approach to care should begin at the time of diagnosis and promote early interventions for patients and family members that match their needs and wishes, address comfort measures, and align with goals of care.^9^ Advance care planning (ACP) is an element central to the palliative approach. It is an ongoing process of discussing preferences and making care plans between healthcare providers and patients, in anticipation of future mental and physical decline.^10^ The implementation of ACP has been suggested to be an effective method for patients to mitigate existential distress while conveying their wishes and improving the patient–provider communicative experience.^11,12^ Newton *et al.*^12^ found that a higher percentage of patients achieved their preferred priorities of care if ACP documentation was initiated by a district nurse in comparison to when it was initiated by palliative care hospital staff, specialist care, hospice, or nursing home care teams.

Although evidence suggests that implementing the palliative approach in primary care is advantageous, various barriers to implementation exist.^13^ Formal training on the palliative approach to care is not consistently incorporated into medical school curricula, thus physicians may not have ready access to evidence-based guidelines and resources and need to hunt for the necessary knowledge and practice skills.^14^ Primary care providers (PCPs) provide first-contact services for patients, and can include team members in addition to general practitioners, such as physician assistants, nurses, nurse practitioners, and district nurses.^15,16^ PCPs are positioned well within healthcare systems to develop ongoing trusting relationships with patients while coordinating and providing personal end-of-life care.^10^ However, the health system may not always support them taking time in their practice routines to develop trusting relationships with patients.^17^ A barrier to care coordination may be less than optimal intra-team communications.^18^

In addition to intra-team communication, advance care plans, and palliative approaches to care in general, often necessitate intersectoral communications between primary care and other health and social agencies. These communications can facilitate care that is consistent with patient preferences, and preferences of health professionals, specialists, nurses, psychologists, counselors, social workers, community workers, and volunteers, because they can assist patients with identifying and accessing critical community-based resources. Increasing the occurrence, efficiency, and effectiveness of such communications is imperative. Despite the understanding that communication between sectors is important, little is known about how sectors communicate with each other. For example, studies have looked into communication with or among different medical areas (i.e. oncology and primary care), however, little is known about how primary care communicates with community sectors.

To better understand these communication channels, this scoping review was guided by the following research question: how do PCPs communicate and enact care plans for older adults with life-limiting illnesses across health, social, and community sectors? Specifically, we wanted to identify the various care plans, forms of communication, and communication pathways shared between PCPs and other health, social, and community sectors.

## Methods

A scoping review aims to map the existing literature in a particular field or topic to understand what types of knowledge or evidence exist. It is intended to incorporate a broad array of sources to understand the depth of existing evidence from the research and gray literature.^19^ This type of review is of particular use for research questions that have not yet been extensively reviewed, are complex, or heterogeneous in nature.^20^ A scoping review provides a rigorous and transparent methodology for mapping the extent of the evidence available. Our interest in exploring communication across health, social, and community sectors fits well within the purview of a scoping review.

### Database search of the existing literature

To conduct the scoping review, a comprehensive search of the existing literature was carried out in collaboration with an academic librarian. The database search was conducted in MEDLINE (ovid), CINAHL (EBSCO), EMBASE (Elsevier), PsychInfo (EBSCO), and Scopus in June 2021. Articles were retrieved from database inception—June 2021. Sample search terms used for the CINAHL search are in Appendix 1.

To be included, studies had to:

a. Be written in English,
b. Focus on how PCPs create, communicate, and enact plans,
c. Be about communicating or creating care plans for older adults with chronic illness,
d. Describe communications between health, social, and community sectors,
e. Be specific to older adults or mention age range 50 or older,
f. Be either theoretical (e.g. best practices for creating and communicating care plans), empirical (e.g. protocol, evaluation), text and opinion papers, or reviews.

Studies were excluded if they

a. Discussed communication within primary care (i.e. primary care physician with primary care nurse) or from hospital to primary care (i.e. discharge planning).
b. When study protocols or studies detailed training for health professionals.

Once the search was completed records were downloaded into Covidence (a review management software system) and duplicates were removed. Covidence was then used to organize the screening and extraction for the review. Reviewers scanned the same 50 articles to validate the inclusion process then met to review their results. There were two stages to the review process. The title and abstract phase focused on identifying articles that reflected key study concepts and the full-text stage explored whether key concepts are operationalized in a way that could address the research question.

A team of five research members independently scanned the title and abstract of every record retrieved, with at least two reviewers per article (JL, NJ, TH, EK, and GW) Potentially relevant papers were retrieved in full and their citation details imported into the Covidence software. Next, two of three independent reviewers (JL, NJ, and TH) assessed the full text of included articles in detail against the inclusion criteria. In addition, reference lists in the included articles were reviewed to identify additional relevant literature. Data were extracted by two independent reviewers (JL and NK). Data were extracted using the Covidence software and pre-coded data extraction categories, see Appendix 2 for detailed template. When conflicts arose during the screening and extraction processes a senior team member with substantial background in the review topic (GW) resolved them. The group then would have a discussion on areas of conflict to ensure all team members had the same understanding of what articles to include and what data should be extracted.

A 2020 Preferred Reporting Items for Systematic Reviews and Meta-Analyses (PRISMA) flow chart was created as a visual summary of the number identified through database search and other sources, as well as the number of records included and excluded.^21^

### Data analysis

Evidence from the search strategy on intersectoral communication between primary care and social or community services regarding older adults was synthesized. Data analysis was completed in five phases: data reduction, data display, data comparison, conclusion drawing, and verification.^22^ All data were combined to provide one complete data set for analysis and cleaned by one author (JL). Data were condensed and coded thematically by a single author (JL) and checked for consistency by a second author (NJ). The extracted data were presented in a tabular format that aligns with the study’s objectives. Results were classified under the main conceptual categories (including country of origin, study population, study design, and setting); aim; outcome measures; communication pathway; type of communication; communication tools; and key findings and implications.

### Data evaluation

Given the focus of this scoping review on mapping existing literature, the Tomlin & Borgetto’s^23^ classification of research pyramid was used to assess the level of evidence. This pyramid breaks down articles into four separate categories (descriptive, experimental, outcomes, and qualitative research) to evaluate the level of evidence of studies included from different study designs. Each category describes four levels of evidence from most to least rigorous. Descriptive research is divided into Level 1: Systematic reviews of related descriptive studies; Level 2: Association, correlational studies; Level 3: Multiple case studies (series), normative studies, descriptive surveys; and Level 4: Individual case studies. Experimental research is divided into Level 1: meta-analyses of related experimental studies; Level 2: individual (blinded) randomized controlled trials; Level 3: controlled clinical trials; and Level 4: single-subject studies. Outcome research is divided into Level 1: meta-analyses of related outcomes studies; Level 2: pre-existing groups’ comparisons with covariate analysis; Level 3: case–control studies; pre-existing groups’ comparisons; and Level 4: one-group pre–post studies. Finally, qualitative research is divided into Level 1: meta-synthesis of related qualitative studies; Level 2: group qualitative studies with more rigor (a, b, and c); Level 3: group qualitative studies with less rigor—(a) prolonged engagement with participants, (b) triangulation of data (multiple sources), and (c) confirmation of data analysis and interpretation (peer and member checking); and Level 4: qualitative studies with a single informant. As part of the review, we included non-studies, such as commentaries and opinion papers identified in our search strategy. Given there is no consensus on how to evaluate these articles, we used Enago’s Academy article^24^ to help categorize and assess these articles.

## Results

**Figure 1.**
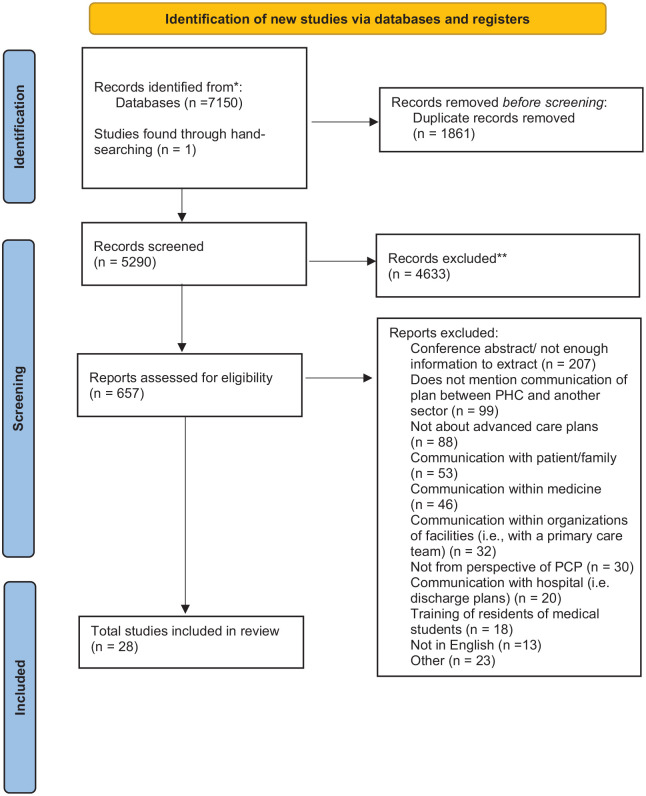
Review process.

The database search of existing research literature identified 27 sources that discussed intersectoral communication within a primary care setting and another health, social, or community sector. There was an additional article found through hand-searching references of included articles, resulting in 28 articles reviewed. These articles discussed goals of care and were targeted toward a more senior population. Search results are in the PRISMA diagram of Figure 1.

### Records identified

The sources involved any form of communication between primary care and another health, social, or community sector about goals of care and referral of older adults to community resources. All 28 articles were published from 1998 to 2021, with a wide variety of geographical locations, including United States (9), Australia (6), United Kingdom (7), Canada (2), Denmark (2), the Netherlands (1), and Norway (1). Thirteen articles specifically discussed the use of electronic health records (EHRs) and how those could facilitate, or in one case hinder, communication across sectors. The study methods varied with the most common being qualitative research (8), text & opinion papers (6), mixed methods (4), quantitative (4), case report (1), and other (4). The communication pathways ranged from primary care communicating directly with community (12), primary care with disease management teams (1), all communication across different sectors (6), primary care with pharmacy (2), primary to patient portal (2), and community providers with other providers (4).

A summary of the extracted data from the included articles identified is provided below, more information is provided in Table 1 with more details regarding care plans and communication in Table 2.

**Table 1.** Characteristics of studies that reviewed how care plans were communicated from primary care to another sector.

Study ID and country	Title	Tomlin level of evidence	Study design and study setting	Aim	Who participated in study	Method of data collection
Abel *et al.*^25^ United Kingdom	Reducing emergency hospital admissions: a population health complex intervention of an enhanced model of primary care and compassionate communities	Outcome research: Level 3	Cohort retrospective study Hospital	Evaluate a population health complex intervention of an enhanced model of primary care and compassionate communities on population health improvement and reduction of emergency admissions to hospital	Patients giving cause for concern (people aged ⩾ 95 years; those with dementia; those identified as high risk of admission using the health numeric risk tool; those with stages 4 and 5 chronic kidney disease; those scoring on the Medical Research Council breathlessness scale at 4 and 5; those on telehealth monitoring; nursing and residential home residents; and palliative care register patients) in two medical practices in Frome, Somerset	Retrospective care planning analysis
Abell *et al.*^26^ United Kingdom	Case management for long-term conditions: the role of networks in health and social care services	Descriptive research: Level 3 and qualitative research: Level 3	Mixed methods Primary care	Explore the relationship and arrangements of a number of case management for long-term conditions (CMLTC) services to identify components of a wider network in which they are embedded and upon which their development is dependent	Managers with lead responsibility for case management service in Greater Manchester	Questionnaire followed by interview with managers
Addicott^27^ United Kingdom	Centralization of end-of-life care coordination: impact on the role of community providers	Qualitative research: Level 3	Qualitative research-case studies Primary care	Understand the impact that centralizing end-of-life care coordination in the community had on community providers	Service managers, commissioners, and providers from two large primary care trust regions in England	Interviews
Ahluwalia *et al.*^28^ United States	Barriers and strategies to an iterative model of advance care planning communication	Qualitative research: Level 2	Qualitative research Medical center	Characterize barriers and strategies for realizing an iterative model of advanced care planning patient–provider communication	Physicians, nurses, social workers, and chaplains in internal medicine, geriatrics, hospital/intensive care, and palliative care in a veterans affairs medical center	Focus groups and semi-structured interviews
Anderson *et al.*^29^ United Stated	Generalist palliative care in the California safety net: a structured assessment to design interventions for a range of care settings	Qualitative research: Level 3	Qualitative research Palliative care setting and partners service line	Identify palliative care quality gaps within a range of settings in the California safety net and to develop theory-based interventions to address them	Adviser pairs—one from palliative care and one from a partner service line—from 10 California public healthcare systems conducted assessments at their sites	Site assessment
Anonymous^30^ United States	Leveraging resources improves care for seniors	Non-study	Opinion, report on program evaluation Hospital	Reduce readmission and emergency departments visits by at-risk low-income seniors served by Wishard–Eskenazi Health using the geriatric resources for assessment and care of elders program	No clear participants	Program evaluation of site
Birke *et al.*^31^ Denmark	A complex intervention for multimorbidity in primary care: a feasibility study	Qualitative research: Level 3 and descriptive: Level 3	Mixed methods Primary care	Assess the feasibility of a patient-centered complex intervention for multi-morbidity (CIM) based on general practice in collaboration with community healthcare centers and outpatient clinics	Patients with at least two of three conditions: diabetes, coronary obstructive pulmonary disease, or cardiovascular disease from a large general practice in Copenhagen	Focus groups
Blakeman *et al.*^32^ Australia	Evaluating general practitioners’ views about the implementation of the enhanced primary care medicare items	Qualitative research: Level 3	Qualitative research Primary care	Investigate the issues for general practitioners surrounding the implementation of the EPC Medicare items for health assessments, care planning and case conferencing	30 GPs in the south western Sydney area	Semi structured interviews with GPs
Bliss and While^33^ United Kingdom	Meeting the needs of vulnerable patients: the need for team working across general practice and community nursing services	Non-study	Opinion Community health center	Fostering engagement in community-oriented integrated care and care management	No clear participants	Review of resources needed
Bose-Brill *et al.*^34^ United States	Validation of a novel electronic health record patient portal advance care planning delivery system	Outcome research: Level 3	Pragmatic randomized control trial Clinical site	Determine the impact on frequency and quality of advance care planning documentation	Patients aged between 50 and 93 years with active portal accounts	Chart analysis
Brungardt *et al.*^35^ United States	Use of an ambulatory patient portal for advance care planning engagement	Outcome research Level 4	Quantitative study Primary care	Increase ACP outcomes by engaging older adults through ACP tools, including an electron Medical Durable Power of Attorney (MDPOA) form	Patients who were 65 years and older with an active health portal account, no cognitive impairment, and no MDPOA on file	Engagement with electronic record
Coleman *et al.*^36^ United States	Caring for seniors: how community-based organizations can help?	Non-study	Opinion Community-based organization	Describe how to use a referral system to community-based organizations. Encourage primary care physicians to explore partnerships with community-based organizations	No clear participants	Use of community referral forms
Harrison and Lydon^37^ Canada	Health visiting and community matrons: progress in partnership	Non-study	Opinion Community	Discus how health visitors can influence and support the development of local long-term condition services and the importance of health visitors working with community matron to share their experience and skills for the benefits of both patients and professionals	No clear participants	Description of how ‘health visiting’ is a positive for seniors with long-term conditions
den Herder-van der Eerden *et al.*^38^ Netherlands	Integrated palliative care is about professional networking rather than standardization of care: A qualitative study with healthcare professionals in 19 integrated palliative care initiatives in five European countries	Qualitative research: Level 2	Qualitative Palliative care	Examine how integrated palliative care takes shape in practice across abovementioned key domains: content of care, patient flow, information logistics, and availability of (human) resources and material) within several integrated palliative care initiatives in Europe	Nurses, physicians, physiotherapists, psychologists, social workers, spiritual caregivers, and pharmacists involved in integrated palliative care initiative	Focus groups
Leighton *et al.*^39^ United Kingdom	Evaluation of community matron services in a large metropolitan city in England	Descriptive research: Level 3 and qualitative research: Level 3	Mixed methods Primary care trust	Evaluate community matron services in a large primary care trust	Patients, carers, and GPs involved with community matron services	Interviews and questionnaires with GPs to determine satisfaction
Lowe^40^ United Kingdom	Care pathways: have they a place in ‘the new National Health Service’?	Non-study	Opinion Primary care	Discuss the use of integrated care pathways (ICPs) as tools for ensuing cost-effectiveness and high-quality patient-focused care	No clear participants	Review of practices
Norman *et al.*^41^ United States	Home- and community-based services coordination for homebound older adults in home-based primary care	Descriptive research: Level 3	Quantitative Home care	Describes how non-medical social needs of homebound older adults are assessed and addressed within home-based primary care (HBPC) practices, and to identify barriers to coordinating HBPC for patients	Members of the American Academy of Home Care Medicine	Online survey of members
Ribe *et al.*^42^ Denmark	Several factors influenced general practitioner participation in the implementation of a disease management program	Descriptive research: Level 2	Retrospective cohort study Disease management programs (DMPs)	To describe the participation among Danish GPs in a disease management program	All GPs in the Central Denmark region with listen patients with chronic diseases	Participation in a chronic care program
Rogers *et al.*^43^ Australia	The advance care planning nurse facilitator: describing the role and identifying factors associated with successful implementation	Qualitative research: Level 3	Qualitative research Primary care and acute hospital	Articulate the components of the ACP nurse facilitator model and identify factors associated with successful implementation	Healthcare professionals who were involved either directly (e.g. trial investigator or nurse facilitator) or indirectly (e.g. professionals who referred patients or provided ongoing care to referred patients) in implementing the intervention	Semi-structured interviews
Gabbard *et al.*^44^ United States	Nurse-led intervention increases advance care planning discussions and documentation	Experimental research: Level 3	Randomized effectiveness trial Primary care	Determine the effectiveness a nurse-navigator ACP pathway combined with a healthcare professional-facing electronic health records interface improved documentation	Primary care patients aged 65 or older who had evidence of multi-morbidity and indication of cognitive or physical impairment or frailty	Nurses’ interaction with electronic health records systems
Rosenthal *et al.*^45^ Canada	Pharmacists’ perspectives on providing chronic disease management services in the community—Part II: development and implementation of service	Qualitative research: Level 2	Qualitative Pharmacy	Report on participants’ suggestion for successful introduction of chronic disease management services provides by pharmacists through education, a model of remuneration, and a plan for implementation	Staff pharmacists, pharmacy managers, pharmacists from the hospital, and primary care settings, regional managers from large-chain retailers	Focus group with pharmacists
Rosstad *et al.*^46^ Norway	Implementing a care pathway for elderly patients, a comparative qualitative process evaluation in primary care	Qualitative research: Level 2	Qualitative research Home care	Investigate the implementation process of the care pathway by comparing the experiences of healthcare professionals and managers in home care services between the participating municipalities	Managers, head nurses, and other staff from ambulant home care services and general practices in six Norwegian municipalities	Focus groups and individual interviews
Rothschild *et al.*^47^ United States	Using virtual teams to improve the care of chronically ill patients	Descriptive research: Level 3	Pilot cross-sectional virtual integrated practice	Describe an interdisciplinary team approach to chronic disease management in the primary care outpatient setting	Physicians, dietitians, pharmacists, and social workers	Piloting different strategies for communication
Rowlands *et al.*^48^ United Kingdom	Improving end-of-life care in the community	Non-study	Program description/evaluation Primary care	Evaluating the Gold Standard Framework (GSF), that enable more people to live well and die well, where they choose	Older adults who wish to die at home	Review of a framework
Shortus *et al.*^49^ Australia	An aged care liaison nurse can facilitate care planning using the enhanced primary care items	Non-study	Opinion/ review of program Primary care	Argue for the removal of barriers to effective coordinated care by offering GPs the services of an aged care liaison nurse	Older adults in a primary care setting	Questionnaires to GPs
Simmons^50^ Australia	Population-based approaches to the integration of primary and secondary care	Descriptive research: Level 4	Case report Chronic care management	Describe how care-mapping was used to develop a ‘population-based integrated care’ approach to diabetes incorporating the tools for integrating the services involved in primary and secondary care	Health workers from local diabetes services, GPs, registered nurses, district nurses, pharmacists, local patients, cultural workers	Review of care maps and referral processes
Swerissen *et al.*^51^ Australia	Community health and general practice: the impact of different cultures on the integration of primary care	Descriptive research: Level 3	Descriptive study Community health centers and general practice	Examine the existing relationship between community health centers and general practice divisions	Chief executive officers or community health centers and executive offices of divisions	Surveys
Tan *et al.*^52^ Australia	GP and nurses’ perceptions of how after hours care for people receiving palliative care at home could be improved: a mixed methods study	Descriptive research: Level 3 and qualitative research: Level 3	Mixed methods Primary care	Investigate the gaps in care from the perspective of general practitioners and palliative care nurses	GPs and palliative care nurses in both urban and rural areas in Australia	Questionnaires

ACP, advance care planning; CIM, complex intervention for multi-morbidity; CMLTC, case management for long-term conditions; DMP, disease management program; EPC, Enhanced Primary Care; GP, general practitioner; GSF, Gold Standard Framework; HBPC, home-based primary care; MDPOA, Medical Durable Power of Attorney.

**Table 2.** Communication plans and tools of studies that reviewed how care plans were communicated from primary care to another sector.

Study ID	Definition of care plan	Type of communication	Communication pathway	Tools to help communicating plan	Patient population description
Abel *et al.*^25^	Patient-centered goal setting	Discharge summaries and a phone visit as a follow-up	Primary care communicating with community health	Health connections Mendip	Frequent users of hospital services
Abell *et al.*^26^	A primary care service dependent on local health and social care services for those with complex long-term needs, they included primary care services, acute/foundation services, adult social care services an intermediate care service	Joint access to computerized client record systems; case managers have access to hospital patient records; multidisciplinary locality meetings; via a designated person; shared assessment documents within or outside the single assessment process; shared review documents; single case file; exchange of written information; patient-held records	Community nurses communicating with GP’s and social workers	Case management for long-term condition (CMLTC) services, multidisciplinary team meetings and shared assessment documents	Individuals accessing primary care trusts in Greater Manchester
Addicott^27^	A centralized administrative center that facilitated a coordinated care package, including telephoning multiple care providers, confirming availability, and communicating the care package arrangements to the particular patient and/or carer	Telephone	Community nurse communicating to administrative center to be relayed onto other care providers (including specialist nurses, community nurses and healthcare assistants, GPs, and personal care providers)	Centralized communication system (telephone)	Any patient in two primary care trust regions in England
Ahluwalia *et al.*^28^	Advance care plan	Across settings and providers in veterans’ affairs across the continuum of care	Communication across health perspectives (i.e. primary care with social work)	Increasing policy attention and financial incentives	Individuals who are nearing end of life and accessing medical services
Anderson *et al.*^29^	Integration of palliative care core elements into usual care provided by services that frequently care for patients who have serious illnesses	Advisors	Palliative care communicating with partner service line	Identifying patients that needed the service, documentation, electronic health records, and an understanding of scope/ role	Individuals receiving specialty palliative care
Anonymous^13^	Evaluation in the home by a nurse practitioner and social worker who report back to a multidisciplinary geriatric team to develop a treatment plan. The plan is shared with the patient’s primary care provider who can make changes and ultimately has final approval	Telephone or in-person	Nurse practitioner/social worker to hospital geriatricians to primary care	Telephone supplemented by the relationship and communications with NPs and social workers	Those with advanced healthcare needs
Birke *et al.*^31^	Care plan included (1) the patient’s chronic conditions, (2) the patient’s care goals, (3) a coordinated care plan with telephone follow-up and future appointments, (4) a plan for medication review in selected patients, (5) potentially shifting hospital outpatient clinic visits to general practice, and (6) referral to community-based rehabilitation and, if needed, home care	Communication of the plan varied	Primary care (GP or nurse) communicating with the community	Creating teams that work cohesively together and use of care manager	Individuals accessing the hospital and have two or more chronic conditions
Blakeman *et al.*^32^	Health assessments, care planning, and case conferencing for people with a chronic illness and multidisciplinary needs	Phone	Primary care communicating with community	Systematically incorporating multiple sectors	Patients of selected GPs
Bliss and While^33^	Anticipatory care	Communicating with community matrons	General practice and community nursing services	Community Matrons	Vulnerable populations
Bose-Brill *et al.*^34^	ACP	Chart communication	Medical professions communicating with patient portals	Auto alerts	Patients 50 years or older and are presenting for a preventive health or chronic disease
Brungardt^35^	Medical Durable Power of Attorney (MDPOA) form to provide educational material about ACPs and enable legal appointment of healthcare decision making	Electronic portal messaging and mail	Primary care communicating with an online patient portal	Online and mail-out services	Elderly patients of primary care clinics in Colorado
Coleman *et al.*^36^	Community organization to aid in care or medical or social programs	Referral letter or note	Primary care communicating with community services	Maintaining two-way communication, early in their collaboration, both parties need to consider who on their staffs will be primarily responsible for communicating with the other side	Seniors trying to access community services
Harrison and Lydon^37^	Plan to get health visitors supported by a community service to patients who may need help or check-ins	Not stated	Primary care communicating with community matrons	Trust and ensuring transparency in understanding role	Older adults with long-term conditions and chronic ill health
Herder-van der Eerden^38^	Integrated palliative care aims at improving coordination of palliative care around patients’ anticipated needs defined as: ‘bringing together administrative, organisational, clinical, and service aspects of palliative care in order to achieve continuity of care between all actors involved in the care network of patients receiving palliative care’	Electronic systems, phone, face-to face, personal notes	Primary, secondary, tertiary, psychology, pharmacy, social	Trusting personal relationships between healthcare professionals, daily transmissions, weekly team meetings	Patients involved with integrated palliative care initiatives
Leighton *et al.*^39^	Plan to support people with complex health needs to maintain optimum health through health promotion, medication titration and self-care, and to support rapid, planned intervention in primary care in the event of health deterioration	Care coordination	Primary care communicating with community matron	Multidisciplinary team	Individuals who have had experience with community matron services
Lowe^40^	Patient need is central to development of pathways the goal is to reconcile the needs of purchaser, providers, professional, and patients. Pathway development commences with the principle that practice should optimize therapeutic effect and aid communication between agencies, professionals and patient/ users to facilitate safe, quality care, and optimize time available to deliver that care	Shared electronic records and care pathways	Communication across primary, secondary and community care	Computerized system that can code to aid in creating pathways	Individuals accessing healthcare services and the pathways they may follow to access these healthcare services; however individual care plans are still tangible and well documented in these pathways
Norman *et al.*^41^	Improving home-based primary care (HBPC) practices in the US understanding, assessment and coordination of patient social needs. To determine the most salient barriers HBPC providers encounter in the coordination process, and whether those barriers impact coordination	Home-based programs	Primary care communicating with non-medical homes and community-based services	Having a point person in the practice that acts as a liaison	Medically complex vulnerable older adults, who are within the home-based primary care
Ribe *et al.*^42^	Chronic care management using various methods	Not stated	Primary care communicating with disease management programs	Not stated	Persons in the central Denmark region suffering from three chronic diseases (diabetes, chronic obstructive pulmonary disease, and acute coronary syndrome)
Rogers *et al.*^43^	Advance care plan	Nurse facilitators (one in the metropolitan area and one in a rural community)	Primary care to community nurses and secondary specialists	Nurse facilitator	Patients with severe respiratory disease at high risk of death in the next 12 months
Gabbard *et al.*^44^	An ACP electronic health record interface to allow for a standardized plan	Electronic records	Primary care nurse with other practitioners	Nurse navigator	Vulnerable older adults
Rosenthal^45^	Chronic disease management provided by pharmacists	Getting pharmacists more involved in counseling patients and problem solving with patients	Primary care communicating with pharmacy	Mentoring period with pharmacist and an appropriate remuneration model	Individuals with multiple chronic disease that are managed with medication
Rosstad *et al.*^46^	Three days after discharge, a home care nurse performs a thorough and structured assessment followed by a consultation with the GP 14 days after discharge. A nurse or nursing assistant performs an extended assessment during the first 4 weeks. A daily care plan is continuously updated, and patient declines are communicated to others by the nurse	Electronic health records	Home nurse to hospitals and GP	A series of flow charts and pathways for people to follow, as well as specific checklists for nursing staff to use	Elderly patients who were in need of home care services after discharge from hospital
Rothschild *et al.*^47^	Virtual integrated practice (VIP) teams work together to develop explicit patient care goals in a specific clinical problem area. Unlike the usual referral process, in which the focus is on a single patient, VIP teams develop population-based goals for patients with the target condition. VIP consists of four strategies: planned communications, process standardization, group activities, and patient self-management	Synchronous (telephone, conference calls, instant messaging) and asynchronous (fax, e-mail, and voice mail)	Primary care communicating with pharmacy, social, and diet healthcare workers	Not stated	Individuals who have Type 2 diabetes, chronic obstructive pulmonary disease, or urinary incontinence
Rowlands^48^	Proactive planning of care, and where possible, delivery of care in alignment with a person’s wishes	Communication amongst a team of care providers	Community (district/community nurses) to other care providers	Establishing a solid line of communication so individual carers do not feel isolated	Individuals who are ‘approaching end of life’, that is, they are likely to die within the next 12 months
Shortus *et al.*^49^	Plan based on patient home assessment that made recommendations to the GP and liaised with relevant care providers	Case coordination	Primary care (practitioner and nurse) communicating with community and other care providers	Aged care liaison nurse aided in helping GP determine patient’s needs, identify services required, prepare care plan, and understanding local services	Older Australians with chronic and complex illnesses
Simmons^50^	Use of a modeling tool for analyzing, documenting, and improving complex business processes to allow for common activities to be identified and linked	Case-mapping	All aspects of a healthcare system (hospital, secondary, primary, and community)	Having an individual act as a broker between practices	Individuals living with complex diabetes
Swerissen^51^	To optimize the working relationship among GPs and between GPs and the wider health system	Joint relationship and working together in regard to planning	General practice communicating with community health centers	Having a strong cooperative relationship, an individual acting as a broker between the two practices	Individuals accessing primary care
Tan *et al.*^52^	After hours home-based palliative care	Palliative care referral system	Primary care with community nurses (palliative nurses)	Standardized written protocol and individual patient protocol	Individuals receiving palliative care at home

ACP, advance care planning; CMLTC, case management for long-term conditions; GP, general practitioner; HBPC, home-based primary care; MDPOA, Medical Durable Power of Attorney; NP, nurse practitioner; VIP, virtual integrated practice.

### Summary of included articles

#### Thematic analysis

Information from this review was sorted into three themes: (1) the importance of relationships across sectors, (2) the importance of community navigators in sharing the care plan and goals of care, and (3) and creating comprehensive and multidisciplinary care plans. The breakdown of all studies and which ones correspond with certain themes is detailed in Table 3.

**Table 3.** All included studies and their corresponding themes.

Study ID	Theme 1: the importance of relationships across sectors	Theme 2: the importance of community navigators in sharing the care plan and goals of care	Theme 3: creating comprehensive and multidisciplinary care plans
Abel *et al.*^25^	✔		
Abell *et al.*^26^			
Addicott^27^			
Ahluwalia *et al.*^28^			✔
Anderson *et al.*^29^			
Anonymous^30^		✔	
Birke *et al.*^31^	✔		✔
Blakeman *et al.*^32^	✔		✔
Bliss and While ^33^	✔	✔	
Bose-Brill *et al.*^34^			✔
Brungardt *et al.*^35^			✔
Coleman *et al.*^36^	✔		
Harrison and Lydon^37^	✔		
den Herder-van der Eerden *et al.*^38^			
Leighton *et al.*^39^	✔	✔	
Lowe^40^			
Norman *et al.*^41^	✔		
Ribe *et al.*^42^			
Rogers *et al.*^43^	✔	✔	✔
Gabbard *et al.*^44^			✔
Rosenthal *et al.*^45^			
Rosstad *et al.*^46^			
Rothschild *et al.*^47^		✔	
Rowlands *et al.*^48^		✔	✔
Shortus *et al.*^49^	✔	✔	
Simmons^50^		✔	
Swerissen *et al.*^51^	✔		
Tan *et al.*^52^	✔		

*The importance of relationships across sectors* Sources reviewed featured a variety of intersectoral communication relationships and pathways. Twelve sources examined communication from health to community sectors^25,31–33,36,37,39,41,43,49,51,52^ and four investigated the opposite direction of communication; community programs communicating to the health sector.^26,27,46,48^ Five sources included communication between health and social sectors,^26,28,30,38,47^ and another four explored interactions between health sectors (primary, secondary, tertiary),^38,40,47,50^ other pathways included primary care to pharmacy^45,47^ and informal care providers.^53^ Communicating between key individuals was considered to be a strong facilitator.

Several sources^24,25,30,31,39,41,44^ emphasized the importance of having strong, foundational relationships between all healthcare practitioners involved in an individual’s care, and suggested that multiple sectors need to be involved in the planning and design of the communication pathway. This collaboration was fostered when there were supportive and trusting relationships^31,48^ or when individuals had defined roles or ‘to-do’ lists.^37,46,51^ To ensure all providers felt part of a team, Rowlands *et al.*^48^ highlighted the importance of making carers aware that there were available supports when navigating patient care. When referring to community services it was important for general practitioners (GPs) and other HCPs to understand what was provided. Aids to understanding community services, as well as promoting effective communication between sectors, described by sources included using standardized letters,^52^ mentoring programs,^45^ specific flow charts,^46^ constant telephone communication,^30^ centralizing communication,^27^ and using shared documents for assessments.^26^ In addition, sources emphasized the transparency of roles.^37^ Anderson *et al.*^29^ found that EHRs were a barrier to communicating plans as they lacked the proper system- wide rollout to be useful and lead to some miscommunication between providers and finding patient history from other providers.

Overall, interpersonal relationships were key to aiding providers in navigating the healthcare system. There is a need to foster communication using practices that help develop interpersonal relationships to better support intersectoral communication. Providers need to look at each other as collaborators to nurture a system that is truly multidisciplinary and becomes the standard of care and best practice.

*The importance of community navigators in sharing the care plan and goals of care* To facilitate communication between health sectors, the importance of an individual that acted as a liaison between sectors, often acting as a broker between various practices, was noted by several sources.^33,43,49–51,53,54^ This figure took on numerous titles; namely, community matron,^33^ nurse navigator,^54^ a broker,^50^ nurse facilitator,^43^ or liaison nurse.^49^ In considering the aforementioned articles depicted the importance of this role and its usefulness from both the perspective of patients and the care team. These healthcare providers aided in communication as they took the burden off the primary provider to find sources, as well facilitating an in-depth discussion with patients about areas they wanted to prioritize. Given community navigators’ extensive experience identifying and reaching out to other community resources they were well equipped to translate between what the PCP wanted and what was available in the community.

In creating and sharing the care plan, community navigators acted as an accessible source to patients, and were tasked with knowing community programs that may be needed to facilitate best care for older patients. It is important for all parties involved (providers, community navigator, family/ friend caregivers, and patient) to work together to better patient care.

*Creating comprehensive and multidisciplinary care plans* Care plans described by the included sources all focused on addressing patients’ goals of care, specifically goals of care nearing end of life. Eight studies incorporated ACP,^28,31,32,34,35,43,44,48^ with one using an EHR ACP interface to allow for standardized planning.^44^ Sources also focused on providing home-based care, featuring home assessments and interdisciplinary care home visits.^30,41,46,49,52^ In addition, chronic care management was featured in four studies.^31,32,42,45^ Important to most care plans was the inclusion of multiple sectors into a single care plan, this entailed a plan that thought of the patient from multiple viewpoints and points of care. This often-involved collaboration between primary and community sectors, aiming to provide patient-centered care. Other plans included support for surrogate decision-makers,^35^ patient-centered goal setting,^25,31,47^ and virtual integrated practices.^47^ Proactively implementing care plans was crucial, and it was suggested that this planning be completed early in the disease trajectory.^32,46^ Emphasis was placed on the importance of effectively and accurately identifying patients that needed, or were expected to need, comprehensive care.^29^ Comprehensive care entailed using a multi-level lens and enrapturing multiple healthcare viewpoints, being part of a multi-dimensional practice could add in this practice^29^ as well as triaging and calling on community resources if needed.^36^

When the care plan was comprehensive and multidisciplinary, patients viewed their entirety of care from a team-based and holistic perspective. As well, the burden of creating a plan was lessened when multiple sources contributed to what needed to be included in the plan.^49^ These comprehensive plans often encompassed patients’ desires and goals of care.

## Discussion

The aim of this review was to examine the communication between primary care and other health, social, or community sectors caring for older adults. Specifically, we wanted to identify the various care plans, forms of communication, and communication pathways shared between PCPs and other health, social, and community sectors. To do this, we synthesized literature from a wide variety of empirical, clinical, and theoretical sources to help gage our understanding of intersectoral communication. The themes identified from this review were relationships, community navigators, and comprehensive care plans.

Numerous intersectoral communication pathways were identified, illustrating the importance of maintaining open, trusting, and communicative relationships among interdisciplinary team members in caring for older adults. A significant communication pathway explored was between health and community sectors. Community-based palliative care is a growing field, and has been suggested by researchers to be the natural evolution of palliative care.^55^ As such, research examining this communication pathway is helpful to the development of comprehensive palliative care in the future. Various tools were identified that assisted in fostering these intersectoral relationships, including mentoring programs, flow charts, and shared assessment documents, as well as constant telephone and centralized communication. EHR in theory would allow for a greater uptake of communication and a greater use of collaborative care, however in practice there has been struggles with integration,^56^ alert fatigue,^57^ and potentially time-consuming training.^58^ Identified barriers to using EHRs is that they have high initial costs, require a change in work habits, may result reduce productivity in the initial stages and require technical knowledge.^59^ Other research has suggested that differences in communication training between healthcare professions can be a barrier to effective relationship-building, citing how the highly descriptive form of communication taught to nurses often clashes with the succinct style taught to physicians.^60^ Similar discrepancies may exist between different healthcare sectors as well, to which training programs, standardized tools, and simulations may serve as solutions.^60^

In addition, having an individual act as a community navigator or care coordinator was another theme that emerged, suggesting that systematically integrating this role into healthcare may be critical in achieving successful intersectoral communication and interdisciplinary care. For example, integration was found to be beneficial in facilitating connections and communications between various sectors and patients. The care coordinator role has also been suggested to have positive impacts on pediatric,^61^ orthopedic,^62^ and chronic disease^63^ care in addition to the palliative approaches described in this review. This role is often accomplished by a nurse. One barrier to this may be the availability or cost of nurses fulfilling this role. A possible solution is training other individuals to complete this role. Volunteers running community-based program have been found to be cost-effective and effective for helping community patients navigate the healthcare system.^64^ In a study examining the feasibility and impact of a volunteer-led healthcare coordination project, volunteers assumed the role of navigators in early palliative care, they found this role to be rewarding and meaningful.^65^ Furthermore, clients were highly satisfied with the volunteer program, noting that volunteers assisted them with coordinating access to services and awareness of available community resources.^65^ A similar study found that volunteer initiatives decreased nurse involvement, but did not replace their participation altogether. It was noted that mentorship provided by nurse navigators was critical to volunteers in understanding their role, and support from these nurses was helpful as volunteers learned their scope and boundaries.^64^

The third theme identified consisted of comprehensive care plans. Literature reviewed examined various forms of care plans, including ACP, home-based care, and chronic care management. This wide range of care plans shows the need for early, comprehensive care plan integration of a palliative approach to care that will keep individuals in their community by increasing the supportive care in their community.^66^ As well, this confirms prior literature that care plans need to be communicated to all sectors to ensure patients and family’s needs are met at the end of life.^17^ The inclusion of intersectoral input in these care plans was emphasized by multiple sources in our review.

The use of EHRs that are continually updated and able to be accessed by multiple healthcare providers may help enact a more comprehensive plan for patients. In our review there was an even split between using and not using EHRs to aid in communicating the plan among the care team. Given the positive effects of having an electronic portal system^35^ that could enable uptake from practitioners and patients, this may be a viable option for healthcare systems. However, given the time-consuming education at the upfront of the use of EHRs^28^ it may need to be implemented over time and gradually into the health system and with proper training and pilot testing of their use.

### Limitations and future directions

The review was limited by our search strategy. Given the breadth of work and terminology in this area and the different nuances of care across health, social and community sectors we may have missed some sources. However, our search strategy was broadened by incorporating the services of an academic librarian, who conducted intensive preliminary searches to refine our final search terms. Our inclusion criteria were limited to studies published in English; therefore, our review may have missed studies in other languages that could have contributed different perspectives to our findings.

In our search, we included commentaries and opinion pieces. Although we did not do a gray literature search, we did conduct a preliminary search of Canadian websites (i.e. private, national, provincial, health authority) to identify existing communications to the public regarding ACP. This search resulted in 56 documents. Most were informational and/or provided guidance to the public on beginning ACP that were linked to region-specific resources for carrying out ACP. These documents did not focus on communications across sectors. It would be beneficial for future reviews to consider a comprehensive gray literature search to identify existing programs that target communication among sectors and community resources not represented in published sources. This search may necessitate contacting community agencies for more in-depth details. Unfortunately, often community programs do not have adequate resources to do program evaluations or to disseminate findings.

## Conclusion

The current scoping review offers a detailed analysis of communication plans across primary care and other sectors. We identified three major themes regarding how to enact and communicate care plans to enhance goals of care in patients. Subsequent evaluation research is needed to understand whether these tools work in practice or can be used on a larger scale. This review illustrates the need to disseminate care plans to all sectors and how additional supports (i.e. community navigators, comprehensive plans, and EHRs) can aid PCPs when working with their patients to achieve their goals of care. It is important that all sectors are aware of the patients’ goals of care and have a strong communication networks. The further development and testing of models that aid healthcare professionals with communicating care plans is needed to ground care plans, so that, they are best practices for patients.

## Appendix 1

**Table:** CINAHL search strategy, May 14, 2021.

#	Query	Results
S1	(MH “Primary Health Care”) OR (MH “Physicians, Family”)	82,241
S2	primary W2 (care OR healthcare)	114,566
S3	S1 OR S2	127,043
S4	(MH “Patient Care Plans + ”)	11,515
S5	“care plan*” OR “care goal*”	24,303
S6	S4 OR S5	24,394
S7	(MH “Communication + ”) OR (MH “Data Communications + ”)	474,483
S8	communicat* OR messag*	251,099
S9	S7 OR S8	588,794
S10	S3 AND S6 AND S9	502

## Appendix 2

**Table:** Data extraction template.

Title of paper
Lead author last name
Publication date
Country in which study conducted
Aim of study
Study design
Dates of intervention/study
Population description
Inclusion criteria
Exclusion criteria
Definition of care plan
Type of communication
X sector communicating to Y sector
Electronic health records?
Tools to help with communicating plan
Main outcomes
Key findings
Implications
Limitations
